# Full hydrodynamic reversibility of the weak dimerization of vancomycin and elucidation of its interaction with VanS monomers at clinical concentration

**DOI:** 10.1038/s41598-017-12620-z

**Published:** 2017-10-05

**Authors:** Mary K. Phillips-Jones, Ryan Lithgo, Vlad Dinu, Richard B. Gillis, John E. Harding, Gary G. Adams, Stephen E. Harding

**Affiliations:** 1 AMR Biophysics Group, School of Pharmacy & Biomedical Sciences, University of Central Lancashire, Preston, PR1 2HE United Kingdom; 2 National Centre for Macromolecular Hydrodynamics, School of Biosciences, University of Nottingham, Sutton Bonington, LE12 5RD United Kingdom; 3 Department of Architecture and the Built Environment, The University of the West of England, Bristol, BS16 1QY United Kingdom; 4 School of Health Sciences, University of Nottingham, Nottingham, NG7 2HA United Kingdom

## Abstract

The reversibility and strength of the previously established dimerization of the important glycopeptide antibiotic vancomycin in four different aqueous solvents (including a medically-used formulation) have been studied using short-column sedimentation equilibrium in the analytical ultracentrifuge and model-independent SEDFIT-MSTAR analysis across a range of loading concentrations. The change in the weight average molar mass *M*_w_ with loading concentration was consistent with a monomer-dimer equilibrium. Overlap of data sets of point weight average molar masses *M*_w_(*r*) versus local concentration *c*(*r*) for different loading concentrations demonstrated a completely reversible equilibrium process. At the clinical infusion concentration of 5 mg.mL^−1^ all glycopeptide is dimerized whilst at 19 µg.mL^−1^ (a clinical target trough serum concentration), vancomycin was mainly monomeric (<20% dimerized). Analysis of the variation of *M*_w_ with loading concentration revealed dissociation constants in the range 25-75 μM, commensurate with a relatively weak association. The effect of two-fold vancomycin (19 µg.mL^−1^) appears to have no effect on the monomeric enterococcal VanS kinase involved in glycopeptide resistance regulation. Therefore, the 30% increase in sedimentation coefficient of VanS on adding vancomycin observed previously is more likely to be due to a ligand-induced conformational change of VanS to a more compact form rather than a ligand-induced dimerization.

## Introduction

**Figure 1:**
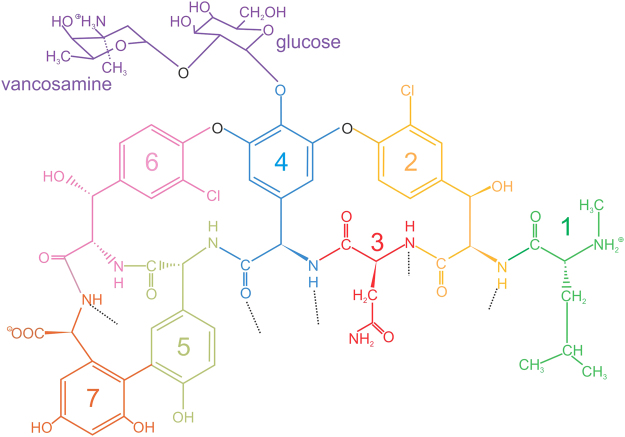
Chemical structure of vancomycin. The disaccharide composed of vancosamine and glucose (purple) is attached at the *para*-position of the phenyl group of (2-[α-4-L-*epi*-vancosaminyl]-β-1-D-glucosyl)-D-phenyl glycine (with residue 4). Also shown are residue 1 (green): N-methyl-D-leucine; residue 2 (light orange): *m*-chloro-β-hydroxy-D-tyrosine; residue 3 (red): asparagine; residue 5 (grey/green): *p*-hydroxy-D-phenylglycine; residue 6 (pink): *m*-chloro- β-hydroxy-D-tyrosine; and residue 7 (dark orange): *m,m-*dihydroxy-L-phenylglycine. Black dotted lines highlight the groups that hydrogen bond with the D-Ala-D-Ala substrate in peptidoglycan. Redrawn from ref.^16^.

Vancomycin (monomer molar mass = 1449 Da) is a member of the glycopeptide family of antibiotics and a ‘last resort’ therapy against life-threatening infections caused by Gram-positive bacteria unresponsive to other antibiotics^1,2^. It is a glycosylated heptapeptide consisting of *N*-methyl-D-leucine, *m*-chloro-β-hydroxy-D-tyrosine (2 molecules), 1-asparagine, *p*-(2-[α-4-L-*epi*-vancosaminyl]-β-1-D-glucosyl)-D-phenylglycine, *p*-hydroxy-D-phenylglycine and *m*,*m*-dihydroxy-L-phenylglycine. The vancosamine – glucose disaccharide is linked to the *para*-position of the phenyl group of the phenylglycine at position 4 in the heptapeptide. The side chains of the heptapeptide backbone are extensively cross-linked including covalent cross-links between pairs of amino acids resulting in a tricyclic structure and a rigid framework with a concave pocket along the peptide backbone^3,4^ (Fig. 1).

Vancomycin and related glycopeptide antibiotics inhibit bacterial cell wall biosynthesis by binding to the –peptidyl-D-Ala–D-Ala peptide of the muramyl pentapeptide of peptidoglycan precursor known as Lipid II. Vancomycin binding has been previously shown to result in inhibition of transpeptidase and transglycosylase activities, affecting the crosslinking process in growing peptidoglycan, formation of glycan chains and incorporation of peptidoglycan precursors leading to osmotic shock and cell lysis^4–7^. Extensive study of how vancomycin binds to Lipid II has been undertaken, yet currently there still remain important questions about the nature of the biologically relevant form of glycopeptide antibiotics. Nieto & Perkins^5^ first reported the aggregation of vancomycin in aqueous solution and these workers used circular dichroism to determine a (molar) dimerization constant *K*_2_ of 800 M^−1^. Dimerisation was confirmed by crystal structural data^8^. NMR spectroscopy studies reported *K*_2_ values for back-to-back dimers of different glycopeptides in solution of 300 - 700 M^−1^ ^9^ and 700 – (6.6 × 10^6^) M^−1^ ^3,10^. However, most studies have focused on the glycopeptide form in the presence of the ligand. For example, using model cell wall precursor peptides such as *N*-acetyl-D-Ala-D-Ala, binding studies in aqueous solution have established that for many glycopeptides, binding is accompanied by increased formation (1-2 orders of magnitude^11^) of asymmetric, back-to-back homodimers^9,12–14^ mediated by sugar-sugar recognition^9,15^. It has been suggested that dimerization enhances the activity of the antibiotic and this is supported by the findings that dimerization and binding of D-Ala−D-Ala *in vitro* are generally cooperative phenomena^3^. For example, dimerization of eremomycin was shown to enhance the affinity of the antibiotic by a factor^3^ of 10. It has been proposed that the dimer binds two adjacent cell wall precursors and that binding to D-Ala−D-Ala at one site on the glycopeptide dimer anchors the dimer to the cell wall and thereby enhances the second binding event through the chelate effect resulting in a stabilised complex^3^. Although vancomycin dimerization is generally considered to be in a back-to-back configuration, crystal structural information of vancomycin in complex with *N*-acetyl-D-alanine revealed the existence of both back-to-back and face-to-face dimers, though the relative importance of each these configurations in antimicrobial action and affinity remains to be established^16^. Molecular dynamic simulations based on NMR data confirmed the possibility of spontaneous formation of both back-to-back and face-to-face dimers and the authors suggested a functional significance for face-to-face dimerization^3^. The crystal structural data also demonstrated higher order oligomerisation for vancomycin in the presence of ligand such as dimer-to-dimer and trimers of dimers, mediated by face-to-face and side-side interactions^17,18^. Size exclusion data provided evidence of hexameric vancomycin in aqueous solution, comprising three pairs of back-to-back dimers^17^.

For clinical use, vancomycin is prepared in 0.9% NaCl and infused at a starting concentration of 2.5–5.0 mg.mL^−1^ in adults so that antibiotic entry proceeds at no more than 10 mg.min^−1^ in order to minimise undesirable side effects of the antibiotic; the aim is to obtain a serum peak level of 25 - 40 µg.mL^−1^ (8 x minimum inhibitory concentration (MIC) value), a trough serum concentration (TSC) maintained at 15–20 µg.mL^−1^ and an initial elevated TSC of 20 µg.mL^−1^ for severe infections^19,20^.

Using the powerful matrix-free technique of sedimentation equilibrium in the analytical ultracentrifuge, the recently available model-independent SEDFIT-MSTAR algorithm and other advanced analysis due to Roark and Yphantis and Kegeles and Rao we now study the reversibility and strength of dimerization of vancomycin in four different aqueous solvents (a) 10 mM HEPES buffer, pH = 7.9, (b) 10 mM HEPES buffer supplemented with 100 mM NaCl, pH = 7.9, (c) 10 mM HEPES buffer supplemented with 100 mM NaCl and 20% glycerol, pH = 7.9 and finally (d) the medically used formulation of 0.9% NaCl (150 mM) referred to above. To maximize sample stability, short solution columns and the Kegeles-Rao method of analysis of the strength of the interactions based on whole-cell weight average molar masses at a low temperature of 7.0 °C were successfully employed.

In addition, in a recent study^21^ the monomeric state in solution of the enteroccocal A-type VanS histidine kinase (HK) was established by sedimentation equilibrium in the analytical ultracentrifuge (confirmed later by SEC-MALS^22^). VanS is a sensor kinase and part of a signal transduction cascade leading to activation of vancomycin resistance genes^21,22^. Sedimentation velocity in the analytical ultracentrifuge (in the presence of 10 mM HEPES buffer supplemented with 100 mM NaCl and 20% glycerol to ensure stability) showed that VanS had an extended conformation in solution, of aspect ratio ~ (12 ± 2). In Phillips-Jones *et al*.^21^ the addition of circa two-fold (in molar terms) vancomycin led to a 30% increase in sedimentation coefficient of VanS. Two possible explanations were provided:

1. *Ligand-induced dimerization*. The addition of vancomycin leads to a dimerization of VanS, or
2. *Ligand-induced conformation change*. The addition of vancomycin leads to a conformation change resulting in a decrease in asymmetry from ~12:1 to about 5:1.

We now ascertain using sedimentation equilibrium which of these explanations (1) or (2) applies, and consider the consequences of this finding, including at clinically-relevant concentrations.

## Results

### Vancomycin self-association

Sedimentation equilibrium in the analytical ultracentrifuge was performed at 47500 rpm (~170,000 g) at a temperature of 7.0 °C in the aqueous solvents (a)–(d) as described above. Analytical ultracentrifugation is a matrix free (no column of separation medium required) technique for the hydrodynamic characterisation of macromolecules. Although vancomycin is too small for the accurate application of sedimentation velocity (formation of a sedimentation boundary from which a distribution of sedimentation coefficients can be obtained) – speeds well in excess of the current limit of 60000 rpm would be required - it is still within the range for sedimentation equilibrium. Sedimentation equilibrium concentration profiles *c*(*r*) vs *r*, where *c(r)* is the concentration at radial position *r* (cm) from the centre of rotation gives a direct measure of molecular weight (molar mass g/mol) unaffected by conformation. For mixtures of more than one component, it gives principally the average molar mass (principally the weight average from concentration distributions recorded using Rayleigh interference optics) either over the whole distribution in the ultracentrifuge cell *M*_w,_ or as a function of radial position *M*_w_(*r*). Because of the small size of the vancomycin and concentration range being studied (0.6 - 10 mg.mL^−1^) no correction for thermodynamic non-ideality was deemed necessary^23^.

**Figure 2:**
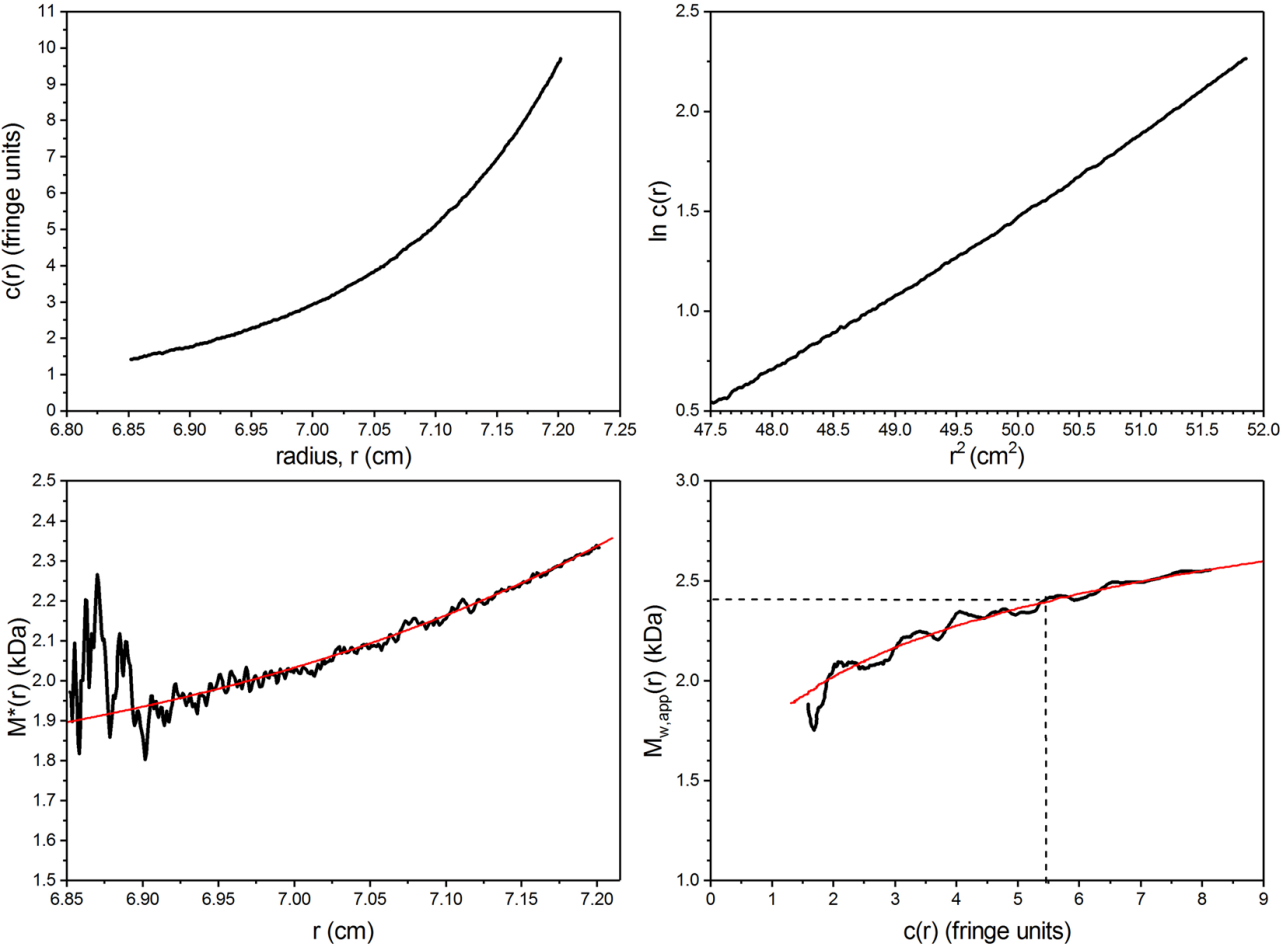
Sedimentation equilibrium SEDFIT-MSTAR^25^ output for analysis of vancomycin. Solvent: 0.9% NaCl at 7.0 °C at a loading concentration of ~1.25 mg.mL^−1^ in conventional (12 mm) path length cells. (**a**) concentration (fringe displacement units) versus radial displacement from the centre of rotation, *r* (**b**) log concentration versus the square of the radial displacement (**c**) extrapolation of the M* function to the cell base to yield the “whole distribution” weight average (apparent) molar mass *M*_w,app_ = (2.4 ± 0.1) kDa; (**d**) plot of the point average apparent molar mass (local molar mass) *M*_w,app_(*r*) – obtained by taking the derivative of the data from plot (**b**) versus local concentration c(*r*) in the analytical ultracentrifuge cell. The value at the hinge point^25^ (where *c(r*) = the cell loading concentration) – dashed line - yields another estimate for the whole distribution *M*_w,app_ ~ (2.4 ± 0.1) kDa. Because of the low molar mass and low concentration, non-ideality effects will be negligible and *M*_w,app_ = *M*_w_.

We employ both the M* method of Creeth and Harding^24^ and the ‘Hinge point’ method^25^ both implemented in the SEDFIT-MSTAR algorithm of Schuck, Harding and coworkers^25^ to obtain *M*_w_ at a series of ultracentrifuge cell loading concentrations *c* (g. mL^−1^). An example is given in Fig. 2 for vancomycin in 0.9% NaCl (ionic strength I = 0.15 mol. L^−1^), at a loading concentration of 1.25 mg/ml and temperature of 7.0 °C. Figure 2a gives the concentration distribution (after correction for baseline effects and the meniscus concentration) and Fig. 2b the corresponding ln *c*(*r*) vs *r* ^2^ plot. Figure 2c shows how the integral function M* function progresses along the *c*(*r*) curve and homes in on the weight average molar mass over the whole distribution *M*_w_: when the cell base is reached (*r* = *b*) the M* function M*(*r* → *b*) = *M*_w_. A value of (2.4 ± 0.1) kDa is returned, a value in excess of the monomer molar mass due to self-association. Figure 2d shows the variation in the point weight average molar masses *M*_w_(*r*) which increases with *c*(*r*) due to self-association. This plot also permits an estimate of the point weight average molar mass at the “hinge point”, *r*_hinge_ the radial position in the cell where the local concentration *c*(*r*) = the original loading concentration *c*, and hence since *M*_w_(*r*_hinge_) = *M*_w_ the weight average molar mass for the whole distribution. A value of (2.4 ± 0.1) kDa is obtained, in agreement with the value from the M* method.

**Figure 3:**
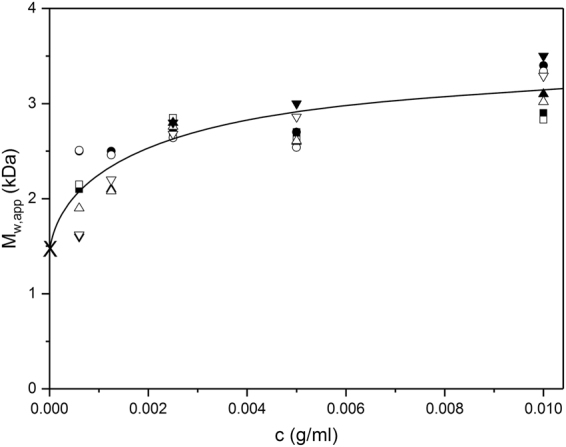
Change of weight average molar mass M_w_ of vancomycin with concentration. From sedimentation equilibrium analysed by SEDFIT-MSTAR for four different solvent data sets. Squares: 10 mM HEPES. Diamonds: 10 mM HEPES + 100 mM NaCl. Up triangles: 10 mM HEPES = 100 mM NaCl + 20% (v/v) glycerol. Down triangles: 0.9% NaCl in deionised, distilled water. Solid symbols – molar masses *M*_w,app_ obtained from M* analysis. Open symbols – molar masses obtained from hinge point analysis^25^. Because of the low molar masses, non-ideality effects can be assumed to be negligible and *M*_w,app_ = *M*_w_. Solid line is a standard French curve fit to the data.

**Figure 4:**
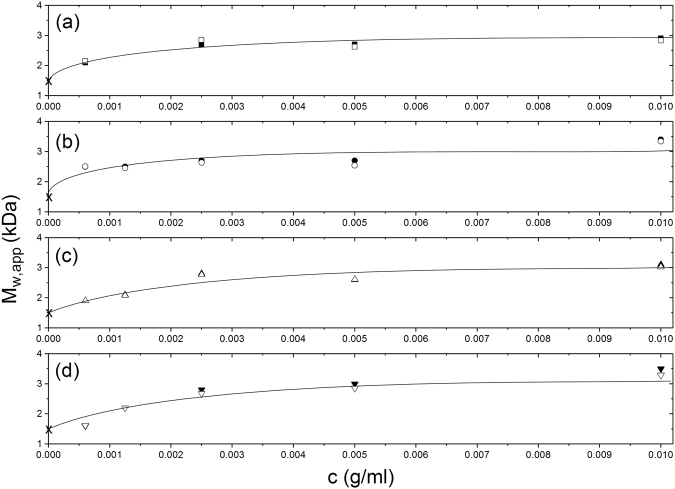
Comparison of the effects of different solvent conditions on the molar mass -concentration behaviour. As Fig. 3 but datasets for each of the solvent conditions shown separately. (**a**) 10 mM HEPES. (**b**) 10 mM HEPES + 100 mM NaCl. (**c**) 10 mM HEPES = 100 mM NaCl + 20% glycerol. (d) 0.9% (w/v) NaCl in deionised, distilled water.

The results are shown for the four different solution conditions combined in Fig. 3, and separately in Fig. 4. The results are all consistent with a monomer-dimer equilibrium extrapolating to a monomer molar mass of ~ 1500 Da (g.mol^−1^), with a suggestion of some further association beyond that above 5 mg.mL^−1^ in the presence of added NaCl.

### Reversibility of vancomycin dimerization

**Figure 5:**
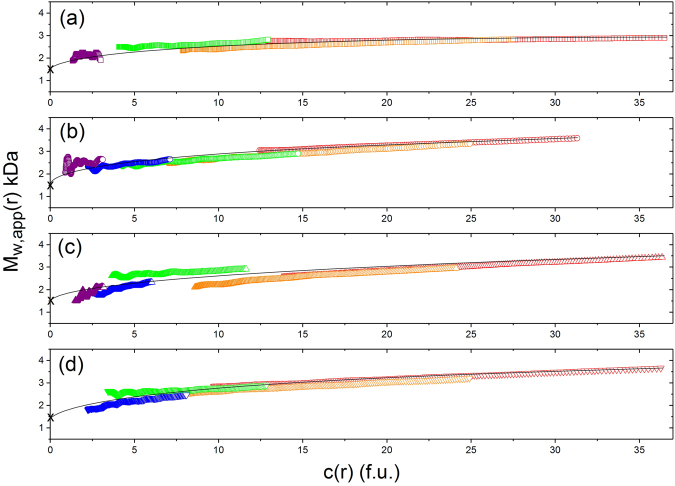
Diagnostic sedimentation equilibrium plots confirming a completely reversible dimerization. Weight average molar mass values *M*_w_(r) at individual radial positions in the ultracentrifuge cell plotted against local concentration *c*(*r*) in interference fringe units for different concentrations: violet (0.625 mg.mL^−1^), blue (1.25 mg.mL^−1^), green (2.5 mg.mL^−1^), orange (5.0 mg.mL^−1^) and red (10.0 mg.mL^−1^). (**a**) 10 mM HEPES. (**b**) 10 mM HEPES + 100 mM NaCl. (**c**) 10 mM HEPES = 100 mM NaCl + 20% glycerol. (**d**) 0.9% NaCl in deionised, distilled water. For a completely reversible self-association the plots should lie, within experimental error on the same curve shown as a standard French curve fit to the data.

The reversibility of the dimerization can be assessed from overlaying plots of the local or “point” weight average molar masses *M*_w_(*r*) as a function of local concentration (in fringe displacement units) *c*(*r*) at individual radial displacements *r* in the ultracentrifuge cell (Figs 2 and 5). For a fully reversible system the profiles must overlay^26,27^ and (allowing for noise at lower concentrations) this appears to be the case for all solvent conditions (a)–(d) studied (Fig. 5). Similar behaviour is seen for example for an electron transfer flavoprotein^28^ and an aminocellulose^29^. Further examples are given in reviews by Harding & Rowe^30^ and Teller^31^ - see also supplementary data of ref.^29^. These contrast for example with the case for a mucin glycoprotein^32^. Of clinical relevance, at 5 mg.mL^−1^ (a clinically important concentration for infusion), ~100% of vancomycin is dimerized (Figs 3–5). As with Figs 3 and 4, the data also indicates that for loading concentrations > 5 mg/mL the association may go further than dimerisation, though higher state complexes have so far only been reported in the presence of binding ligands^4,17,18^.

### Assessment of association/ dissociation constants for vancomycin dimerization

**Figure 6:**
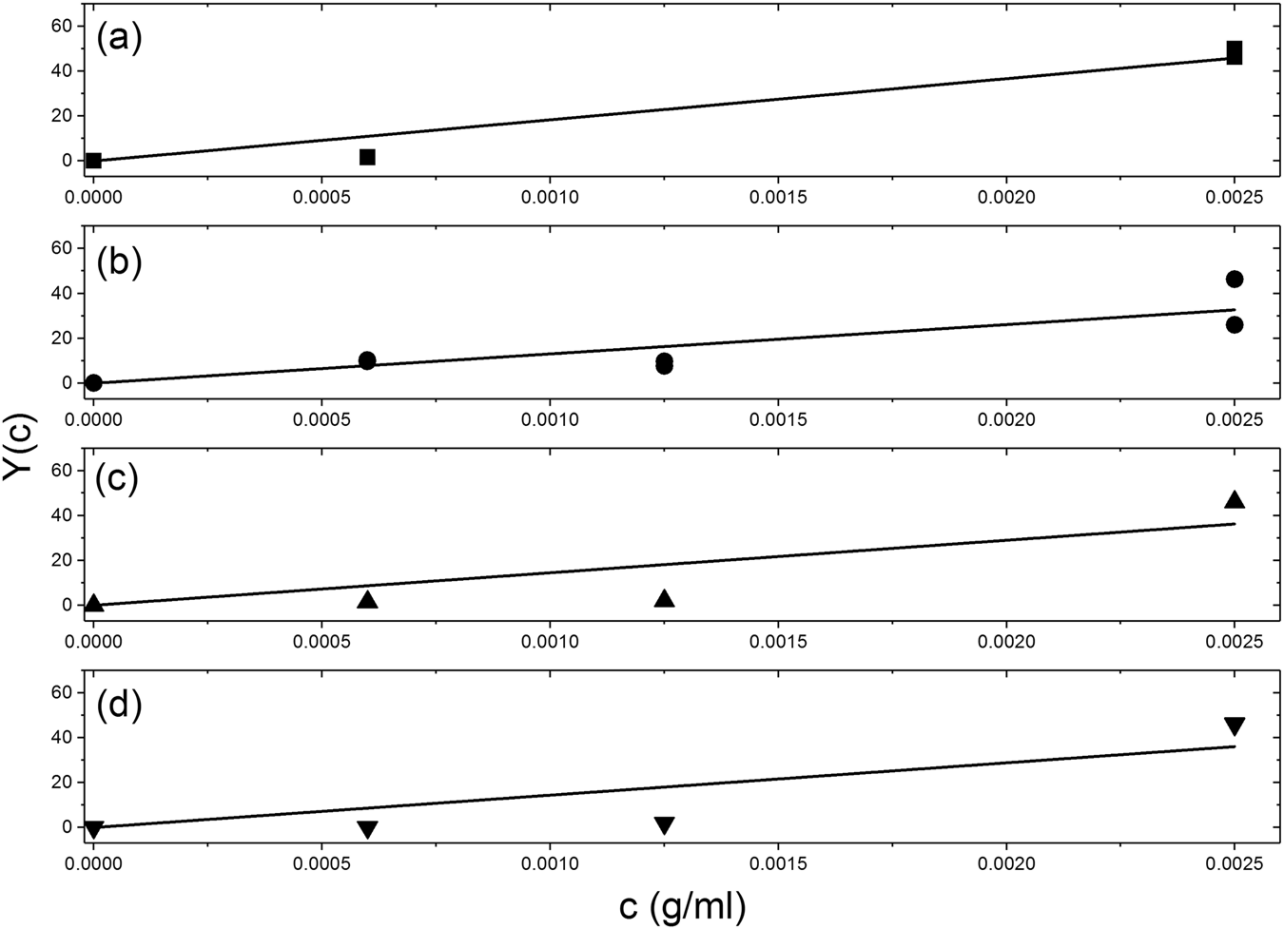
Kegeles-Rao evaluation of the association constants *k*_2_ (mL/g), *K*_2_ (M^−1^), dissociation constant K_d_ (μM) and standard free energy of association Δ*G*° for the dimerization of vancomycin. Least squares fitting to the Kegeles-Rao equation^27,33^ *Y*(*c*) ≡ *M*_1_{*M*_w_(*c*) − *M*_1_}/{(2*M*_1_ − *M*_w_(*c*))^2^} = *k*_2_.*c*. where the *M*_w_(*c*) are the weight average molar masses (averaged over the whole macromolecular distributions) at different loading (**a**) 10 mM HEPES. (**b**) 10 mM HEPES + 100 mM NaCl. (**c**) 10 mM HEPES = 100 mM NaCl + 20% glycerol. (**d**) 0.9% NaCl in deionised, distilled water. Lines shown are obtained by least squares analysis. Values are given in Table 1.

Now that full reversibility has been established we can now assess the strength of the dimerization in terms of the association (dimerization) constant *k*_2_ (mL.g^−1^) or the corresponding molar quantity *K*_2_ (mL mol^−1^ or l mol^−1^ ≡ M^−1^) and molar dissociation constant *K*_d_ (M) = 1/*K*_2_, from the variation of *M*_w_ with *c*. Following Kegeles and Rao^33^ and equation III-66 of ref.^27^, for a fully reversible thermodynamically ideal dimerisation:

1 $$Y(c)\equiv {M}_{1}\{{M}_{w}(c)\,{\textstyle \text{-}}\,{M}_{1}\}/\{{(2{M}_{1}{\textstyle \text{-}}{M}_{w}(c))}^{2}\}={k}_{2}\,.\,c$$

with *M*_1_ the monomer molar mass ( = 1449 Da or g.mol^−1^). Plots of *Y*(*c*) versus *c* for the four different solvent conditions (a)–(d) are given in Fig. 6, and the corresponding values for *k*_2_, *K*_2_ and *K*_d_ for vancomycin dimerization are given in Table 1. For these evaluations we have used the Durchschlag-Zipper method for the evaluation of partial specific volumes from the atomic composition^34^ and assumed this does not appreciably change across the range of ionic strengths studied (0.01–0.15 mol L^−1^) nor for the addition of glycerol. These assumptions are considered below. Corresponding ‘affinities’ or standard Gibbs free energies for dimerization Δ*G*° are also given. All interactions are relatively weak (dissociation constants *K*_d_ ~ 25–75 μM, and free energies of association ~ 23 kJ mol^−1^).

**Table 1:** Values from sedimentation equilibrium for the association constants *k*_2_ and *K*_2_, the dissociation constant *K*_d_ and “affinities” (standard Gibbs free energy change Δ*G*°) for the dimerization of vancomycin in 4 different solvent conditions.

Solvent	*k*_2_ (mL.g^−1^)	*K*_2_ (M^−1^)	*K*_d_ (μM)	Δ*G*° (kJ.mol^−1^)
(a) 10 mM HEPES	18400 ± 5000	27600 ± 7900	35 ± 10	−(23.9 ± 1.1)
(b) 10 mM HEPES + 100 mM NaCl	13100 ± 4000	20000 ± 6000	50 ± 15	−(23.1 ± 0.7)
(c) 10 mM HEPES + 100 mM NaCl + 20% (v/v) glycerol	14500 ± 3200	22000 ± 4000	45 ± 10	−(23.4 ± 0.1)
(d) 0.9% (w/v) NaCl (150 mM)	14400 ± 3600	21000 ± 5200	40 ± 10	−(23.3 ± 0.6)

Temperature = 7.0 °C.

Other studies have reported dimerization constants in the absence of ligand to be in the low millimolar range: 3.9 × 10^3^ M^-1^ (*K*_d_ ~ 250 µM)^35^ and 4.7 × 10^2^ M^−1^ (*K*_d_ ~ 2.1 mM)^11^ and 51 M^−1^ (*K*_d_ ~ 19.6 mM)^36^. Although the lower *K*_d_ values obtained in the present study indicate a somewhat stronger self-association than previously reported, all studies to date (including our own) overall indicate only a weak dimerization, even though dimerization has been considered by some to be clinically important for antibiotic activity^3,4,9,10,14–16^.

### Elucidation of the nature of the interaction of vancomycin with enterococcal VanS

Having established the strength and fully reversible nature of the dimerization of vancomycin we can now elucidate the nature of its interaction with enterococcal A-type VanS, an interaction which may lead to triggering of glycopeptide resistance gene expression. The existence of the interaction was first shown by a combination of hydrodynamics and CD spectroscopy^21^, and like the vancomycin dimerization, was subsequently shown to be weak (K_d_ ~ 70 μM)^22^.

In contrast to the reversible monomer-dimer equilibrium for vancomycin, VanS appears to be purely monomeric in aqueous solution^21^ from sedimentation equilibrium in the analytical ultracentrifuge in the presence of HEPES buffer supplemented with 100 mM NaCl and 20% glycerol (i.e. solvent “c” described above), an observation confirmed by the independent method of SEC-MALS – size exclusion chromatography coupled to multi-angle light scattering^22^. Phillips-Jones *et al*.^21^ also showed using sedimentation velocity in the analytical ultracentrifuge, and under the same solvent conditions, that VanS had an extended conformation in solution of aspect ratio (12 ± 2). They further reported that a 2-fold (on a molar basis) addition of vancomycin led to a 30% increase in sedimentation coefficient of VanS. The dissociation constant of ~70 μM (ref.^22^) would result in no detectable change in the molar mass of VanS as a result of binding of the vancomycin, but the significant shift in the sedimentation coefficient of VanS could be due to either (1) a ligand induced dimerization or (2) a ligand induced conformation change with a decrease in asymmetry (aspect ratio from ~12:1 to ~ 5:1) as noted above and in ref.^21^.

**Figure 7:**
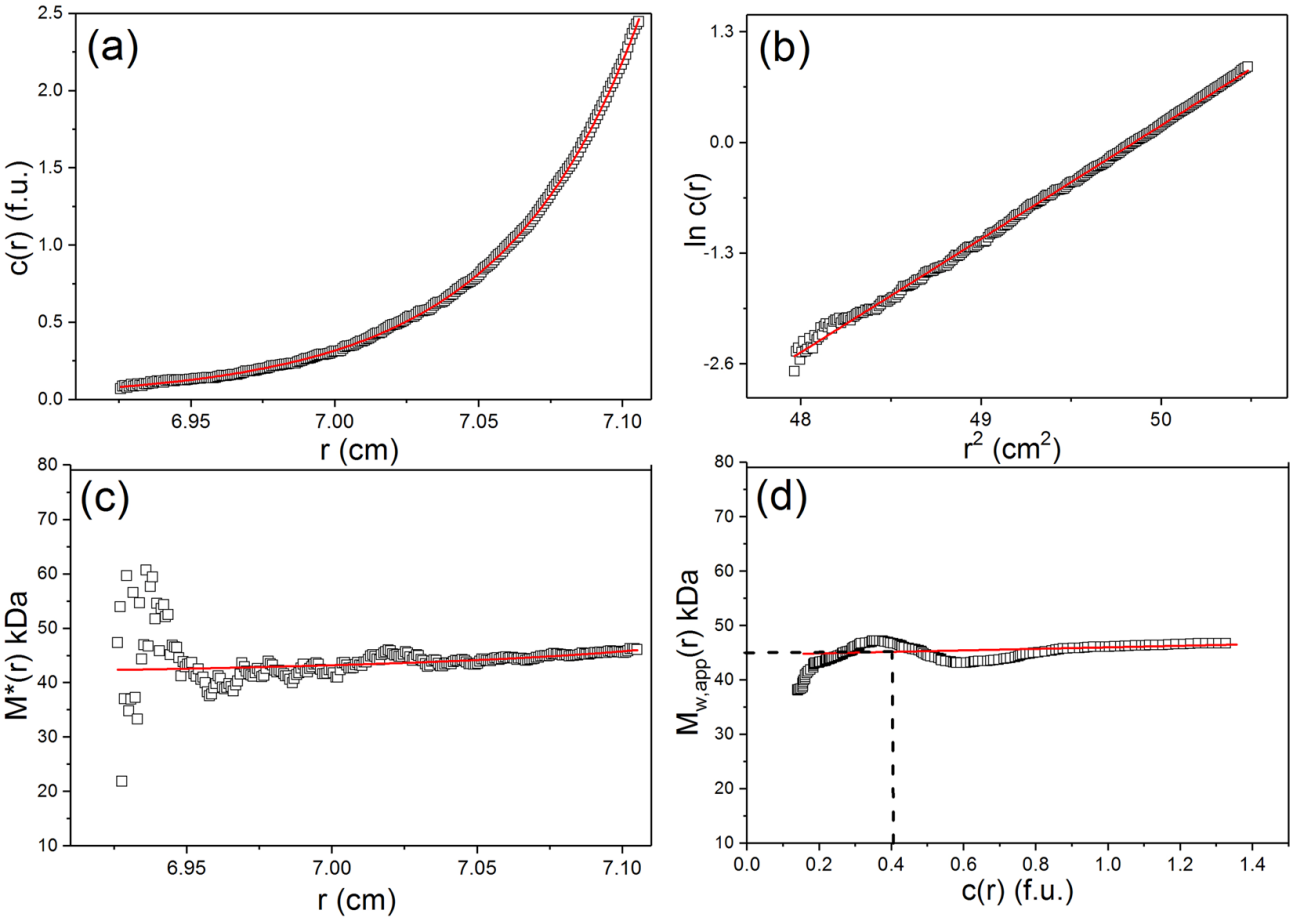
Sedimentation Equilibrium SEDFIT-MSTAR output for analysis of VanS in the presence of vancomycin. Solvent: 10 mM HEPES = 100 mM NaCl + 20% glycerol, pH~7.9, I = 0.1 at 7.0 °C at a loading concentration of ~0.3 mg.mL^−1^ (VanS) supplemented with 19 μg.mL^−1^ vancomycin in long (20mm) path length cells. (**a**) concentration (fringe displacement units) versus radial displacement from the centre of rotation, *r* (**b**) log concentration versus the square of the radial displacement (**c**) extrapolation of the M* function to the cell base to yield the “whole distribution” weight average (apparent) molar mass *M*_w,app_ = (45.0 ± 1.0) kDa; (**d**) plot of the point average apparent molar mass (local molar mass) *M*_w,app_(*r*) – obtained by taking the derivative of the data from plot (**b**) versus local concentration *c*(*r*) in the analytical ultracentrifuge cell. The value at the hinge point (where *c(r*) = the cell loading concentration) – dashed line - yields another estimate for the whole distribution *M*_w,app_ ~ (45.0 ± 1.0) kDa. Because of the low concentration, non-ideality effects will be negligible and *M*_w,app_ = *M*_w_.

To help us delineate between explanation (1) or (2) we now use sedimentation equilibrium to explore the oligomeric state of VanS in the presence of 2-fold vancomycin (19 μg.mL^−1^). This vancomycin concentration is equivalent to the target initial trough serum concentration desired in the clinic of 20 µg.mL^−1^ and the target maintenance level of 15 – 20 µg.mL^−1^ desired thereafter^19,20,37^ and therefore chosen here to investigate bacterial VanS-vancomycin interactions. Figure 7 (VanS with ligand) gives the comparable SEDFIT-MSTAR set of plots to those of Fig. 5 (VanS without the ligand) of Phillips-Jones *et al*.^21^. Figure 7a shows the concentration distribution *c*(*r*) in fringe displacement units as a function of radial position *r*, Fig. 7b the corresponding plot of ln*c*(*r*) vs *r* ^2^: the ~ linear nature of this plot is our first indicator of a highly monodisperse solution. The M* extrapolation (Fig. 7c) and hinge point estimation methods (Fig. 7d) from this algorithm both give values for the overall weight average molar mass for the distribution, *M*_w_ = (45000 ± 1000) Da slightly lower than the values obtained in the absence of vancomycin, and the monomeric value from mass spectroscopy^21^ (*M* = 45765 Da) with no evidence for dimerization. The slightly lower values may reflect the small influence of the 19 μg.mL^−1^ vancomycin on the weight average molar mass values. This can only mean the VanS remains monomeric in the presence of vancomycin and explanation #2 now appears the most plausible explanation of the results.

## Discussion

### Reversible weak dimerization of vancomycin: Proportion of monomers and dimers at administered concentrations

Vancomycin has to be administered at concentrations high enough so as to be effective but also low enough to minimize the risks of damage to human tissue such as thrombophlebitis (deep vein thrombosis) and nephrotoxicity^19,20,38–40^. In infusion formulations administration doses are typically at 5 mg.mL^−1^ or less. This concentration of 5 mg.mL^−1^ would correspond to ~ 100% dimers (Eq. 1; Figs 3 and 4). However, this is substantially diluted in the serum to 15 - 20 μg.mL^−1^, minimizing the risk of toxicity^38,39^, however, the present study has established that the monomeric form of vancomycin predominates at this concentration: the proportion of dimers will be < 20%.

### Monomeric nature of VanS

A previous hydrodynamic study demonstrated that the purified intact VanS histidine kinase occurs as a monomer under the aqueous solution conditions used in the present study^21^. The monomeric nature of the protein was also found to be the case using the alternative approach of SEC-MALS and in the presence of 0.025% n-dodecyl-β-D-maltoside detergent^22^. Classic prototypical HKs are considered to be membrane-bound homodimers that require two cytoplasmic catalytic ATP-binding (CA) domains to achieve *trans*-autophosphorylation (reviewed in ref.^41^). However, a full structural understanding of the autophosphorylation reaction has yet to be elucidated and there are examples of histidine kinases, which deviate from this scheme, including those possessing highly dynamic CA domains. For example, autophosphorylation may occur in *cis*, rather than in *trans*, as documented for the HK853 kinase^41^. So *cis*-autophosphorylation occurs by an intrasubunit mechanism; that is, the ATP bound in the CA domain of one subunit phosphorylates the histidine residue in the same subunit monomer^41,42^. As VanS has been shown in the present study to occur as a monomer in our experimental system, and it has also previously been shown that VanS protein stored in these same conditions possesses strong autophosphorylation activity even in the absence of vancomycin ligand^21^, it is possible that under the conditions of our experiments, VanS performs *cis*-autophosphorylation reactions.

The versatility and accessibility of monomeric VanS in aqueous solvent makes it a highly suitable model system for investigating ligand-induced conformational changes. Under these conditions, monomeric VanS demonstrates weak interactions with vancomycin^21^ with a *K*_d_ value of 70 µM^22^. The finding here of a conformational compaction of the elongated VanS protein in response to vancomycin addition; that is, a decrease in asymmetry from ~12:1 to about 5:1, is consistent with one of the conformational responses expected for a HK during *cis*-autophosphorylation (or indeed *trans*-autophosphorylation), namely, an approach of the C-terminal CA domain towards the phospho-accepting histidine in the central DHp (dimerization histidine phosphotransfer) domain. Further studies are required to confirm whether or not this is the case following VanS-vancomycin interactions and indeed if the situation we observe in aqueous solution occurs similarly within the membrane. It is intriguing that under the conditions used in the present study and previously^21^, that the VanS membrane protein remained soluble in the absence of added detergent and that no detectable detergent remained bound after dialysis prior to AUC analysis and indeed post-purification (as revealed by mass spectrometry)^21^. This may be attributable to the large soluble non-membrane portion (predicted to be ~90%) of this sensor kinase. This feature has been useful (and indeed highly suitable) for our initial AUC-based interrogations (here and in ref.^21^) to characterise VanS conformational changes in simple aqueous solvent. Further studies are now underway to determine whether the VanS-vancomycin interactions observed in aqueous solution here also occurs similarly within the membrane environment.

## Methods

### Source of vancomycin

Vancomycin was obtained from Duchefa Biochemie, Haarlem, The Netherlands. A partial specific volume ῡ of 0.67 mL g^−1^ was calculated from the composition (Fig. 1) and equation 4 of Durchschlag & Zipper^34^ which allows for corrections for covolume, ring formation and electrostriction from charged groups. This value is in good agreement with the value of 0.69 ml g^−1^ of Linsdell *et al*.^35^ based on amino acid and carbohydrate composition. The validity of the Durchschlag-Zipper approach for aqueous systems had been confirmed by a systematic comparison of calculated and experimental partial specific volumes of different classes of organic and biochemical compounds, including small molecules and polymers of ionic nature^34^. We make the assumption that there is no ionic strength (I) dependence of ῡ in the range studied (I = 0.01-0.15 mM) through electrostriction or related effects (see, for example ref.^43^) and no effect on ῡ through the addition of glycerol. Jaenicke and Lauffer^44^ have shown that the ‘apparent’ partial specific volumes for tobacco mosaic virus (TMV) and TMV protein increase by ~ 0.1% and 0.8% respectively (see for example ref.^44^) in 25% glycerol.

Solution concentrations of vancomycin *c* (g. mL^−1^) were determined densimetrically from the relation^45^

2 $$c=(\rho \,{\textstyle \text{-}}\,{\rho }_{o})/(1\,{\textstyle \text{-}}\,\bar{\upsilon }{\rho }_{o})$$

where ρ and ρ_o_ are the solution and solvent densities respectively, measured on an Anton-Paar (Graz, Austria) digital density meter.

### Source of VanS

Purified VanS protein was prepared as described previously^21^ and dialysed into 10 mM HEPES, 100 mM NaCl, 20% glycerol buffer pH 7.9 (HGN buffer). Reaction mixes comprised VanS at a final concentration of 5.6 µM (0.256 mg.mL^−1^) in the presence of either 12.8 µM (0.019 mg.mL^−1^) (final concentration) vancomycin dissolved in 20 mM Tris.HCl pH 8.0 or an equivalent volume of 20 mM Tris.HCl pH 8.0 buffer as control. A partial specific volume of 0.747 mL.g^−1^ was used as previously reported^21^.

### Sedimentation equilibrium in the analytical ultracentrifuge

Sedimentation equilibrium experiments were performed using a Beckman (Palo Alto, CA, USA) Optima XL-I analytical ultracentrifuge equipped with Rayleigh interference optics and an automatic on-line data capture system, to record equilibrium concentration distribution profiles. For the characterisation of vancomycin dimerization, standard 12 mm path length cells with aluminium epoxy centrepieces were used and loaded with matched 100 μL of solution (solution channel) and reference solvent (reference channel). A rotor speed of 47500 rpm was used at a temperature of 7.0 °C. The rotor speed was found to be optimum for giving an appropriate concentration (fringe) increment from meniscus to base (see Fig. 2). Short solution columns – resulting in relatively rapid equilibrium (<48 h) and low temperature ensured the stability of the samples. With the short columns solvent redistribution effects were negligible. Rinde^46^ calculations for the redistribution of glycerol (*M* = 92 Da) show a ratio of concentration at the base to meniscus at equilibrium of ~1.04. Long columns or multiple-speed averaging were inappropriate.

Concentration distributions at sedimentation equilibrium were analysed using the model-independent SEDFIT-MSTAR procedure of Schuck, Harding & coworkers^25^ based on the M* function of Creeth and Harding (1982)^24^. Because loading concentrations up to only 10 mg.mL^−1^ are used, and particularly because of the small size of the vancomycin, non-ideality effects (which tend to lead to underestimates of the molar mass) will also be relatively small and we make the approximation that apparent weight average molar masses *M*_*w*,*app*_ are equal to the true weight average molar masses *M*_*w*_ (ref.^25^).

For the characterisation of VanS in the presence of vancomycin we followed the procedure of Phillips-Jones *et al*.^21^ using the modified long (20mm) optical path length double-sector titanium cells with sapphire windows. These were loaded with 140 µL of solution (containing a final protein concentration of 5.6 µM (260 μg.mL^−1^) in the presence of 12.8 µM (19 μg.mL^−1^) (final concentration) vancomycin as described above), and a matching amount of reference solvent dialysate in the appropriate channels. An equilibrium speed of 25000 rpm was employed at a temperature of 7.0 °C to ensure stability over the long time course (96 hours) of the experiment. The use of long path length cells meant that low loading concentrations could be used to give a sufficient signal (~0.3 mg.mL^−1^) for records to be interpreted. Although VanS is much larger than vancomycin, at such low concentration, non-ideality effects will also be relatively small and we can also make the approximation^23,25^ that the apparent weight average molar mass *M*_*w*,*app*_ is equal to the true weight average molar mass *M*_*w*_.
